# Extracellular Vesicles in the Pathogenesis of Viral Infections in Humans

**DOI:** 10.3390/v12101200

**Published:** 2020-10-21

**Authors:** Allen Caobi, Madhavan Nair, Andrea D. Raymond

**Affiliations:** Department of Immunology and Nanomedicine, Herbert Wertheim College of Medicine at Florida International University, Miami, FL 33199, USA; acaob001@fiu.edu (A.C.); nairm@fiu.edu (M.N.)

**Keywords:** exosomes, extracellular vesicles (EVs), viruses, pathology, HIV, ZIKA, retrovirus, herpes virus, coronavirus, therapeutics

## Abstract

Most cells can release extracellular vesicles (EVs), membrane vesicles containing various proteins, nucleic acids, enzymes, and signaling molecules. The exchange of EVs between cells facilitates intercellular communication, amplification of cellular responses, immune response modulation, and perhaps alterations in viral pathogenicity. EVs serve a dual role in inhibiting or enhancing viral infection and pathogenesis. This review examines the current literature on EVs to explore the complex role of EVs in the enhancement, inhibition, and potential use as a nanotherapeutic against clinically relevant viruses, focusing on neurotropic viruses: Zika virus (ZIKV) and human immunodeficiency virus (HIV). Overall, this review’s scope will elaborate on EV-based mechanisms, which impact viral pathogenicity, facilitate viral spread, and modulate antiviral immune responses.

## 1. Extracellular Vesicle (EV) Biogenesis

**Figure 1:**
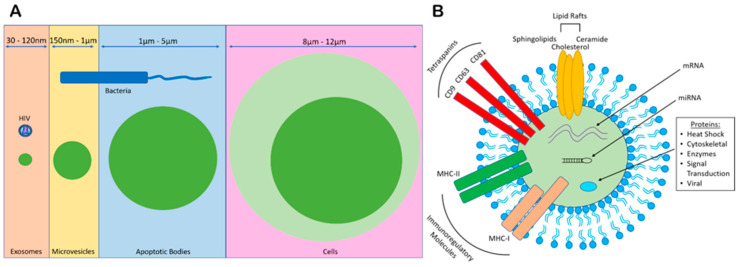
Size ranges of EVs and characterization. (**A**) Exosomes released when MVBs fuse with the PM are vesicles that range from 30–120 nm in diameter. Due to the similarity in size to viruses, exosomes are difficult to isolate from virus-infected blood. Microvesicles ranging from 150 nm–1 µm in diameter derive via shedding/budding from the PM surface. Apoptotic vesicles released from apoptotic cells range from 1 µm–5 µm in diameter. (**B**) Exosomes transport a variety of proteins and genetic material. Lipid raft-derived microdomains form larger domains responsible for inducing budding in an ESCRT-independent pathway of lateral cargo segregation. Exosomes are highly enriched with tetraspanins, which play a critical role in the ESCRT-independent pathway of endosomal sorting and function as exosome-defining surface markers. Depending on the cell of origin, exosomes may contain differing immunoregulatory molecules, such as MHC-I/II. Lastly, exosomes traffic a variety of host cell/viral protein, mRNA, and miRNA.

EVs formation occurs in most nucleated cells and is evolutionarily conserved [1]. Bodily fluids, including saliva, CSF, blood, and urine, all contain EVs. Initially, scientists thought EVs were waste products, but researchers have found that EVs are integral to intercellular communication and signal transduction [2]. EVs are classified as one of the following: apoptotic bodies, microvesicles, or exosomes (Figure 1). Cells undergoing apoptosis release apoptotic bodies 1–5 µm in diameter and are also capable of releasing smaller EVs such as apoptotic microvesicles (<1 µm) [3]. However, it remains unclear if the formation of apoptotic microvesicles occurs under the same mechanisms responsible for microvesicle generation in healthy cells [3]. Unlike apoptotic bodies, microvesicles and exosomes are derived from healthy cells, have been extensively characterized, and are critical in regulating the immune response and intercellular communication [3]. Microvesicles are primarily generated via shedding/budding from the plasma membrane (PM) and are between 150 nm and 1 µm in diameter [4]. Microvesicles can also transport pro-inflammatory miRNAs and cytokines such as IL-1β, thereby initiating the acute inflammatory response and modulating the immune response [4,5]. Unlike microvesicles, exosomes are intraluminal vesicles (ILVs) ranging from 30 to 120 nm in diameter, are formed during the maturation of multivesicular bodies within the late-endosome via inward budding of the endosomal membrane, and are released into extracellular space when a multivesicular body (MVB) fuses with the PM [1,6,7]. However, the ILVs may undergo degradation if the MVB fuses with a lysosome instead of the PM. The process determining the fate of MVB fusion, in which the MVB either fuses with the PM releasing exosomes or fuses with the lysosome for lysosomal degradation, is not yet fully understood. However, it is hypothesized that MVB fate results from inhibition of either pathway as lysosome inhibition results in increased EV release [8].

**Figure 2:**
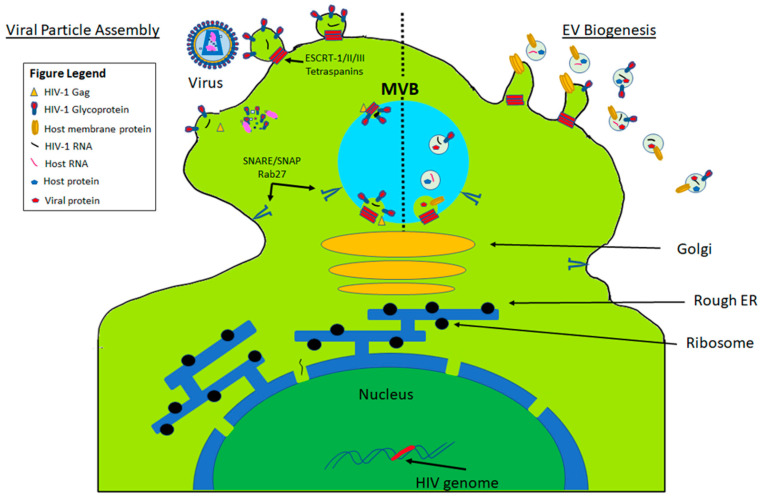
Comparison of viral and EV biogenesis. Infected cells concurrently release EVs and retroviral particles, possessing shared pathways at the MVB and the PM, whilst incorporating SNARE/SNAP Rab27 and tetraspanins, and ESCRT proteins in both pathways [1]. Depicted here is: sorting and transport of the exosome-specific proteins to the nascent ILVs, and excision from the MVB, Gag-mediated virion assembly at the PM or the MVB, and EV and virion release, [1,3].

Bridging of the exosome biogenesis and viral replication/assembly pathways depicts a shared mechanism between EV and virus particles (Figure 2). Virally infected cells release both EVs and new virions simultaneously [1]. Post-infection, viral RNAs in the cytoplasm, interacting with other viral factors undergo Gag-mediated virion assembly at the PM or close to the MVB [1]. EV or virion-containing MVB employs SNARE/SNAP Rab27 to fuse with the PM, releasing both EVs and virions [1]. Exosome-specific proteins are sorted and then transported to ILVs, where exosomes acquire their contents and are excised within the MVB [3]. Mono-ubiquitinated cytosolic domains in these exosome-specific proteins serve as sorting signals to ILVs and capturing sites for the endosomal sorting complex required for transport (ESCRT) [3]. ESCRT-0, ESCRT-I, ESCRT-II, and ESCRT-III compose the ESCRT protein machinery required for sorting the ubiquitinated cargo to the ILVs. This sorting is initiated by the interaction of the endosomal protein’s (Hrs) double zinc finger domain’s binding with phosphatidylinositol 3-phosphate (PtdIns-3-P), permitting ESCRT-0 binding to the endosomal membranes and eventual recruitment cascade of ESCRT-I, which recruits ESCRT-II, resulting in recruitment of ESCRT-III [4]. ESCRT-0 concentrates all non-ESCRT-III proteins that interact with ubiquitylated cargo via their ubiquitin-binding subunits, capturing cargo after it has been concentrated by [6]. The captured cargo is driven into ESCRT-III filament produced invaginations, deubiquitinating the cargo, and excising the cargo-filled ILV [6]. Several reports describe alternative pathways. For example, ESCRT-independent EV generation was observed in oligodendrocytes, where ceramide is released from the breakdown of sphingolipids and promotes domain-induced budding of the ILVs [9]. This proposed ESCRT-independent pathway is dependent on lateral cargo segregation via lipid-raft-derived microdomains, which then merge into larger domains, inducing budding [9]. Inhibition of sphingomyelinases, which hydrolyze sphingomyelin into ceramide, have been observed to inhibit exosome release but promote microvesicle secretion from the PM, showcasing that ceramide is critical for exosome formation [10]. Although EVs share a common biogenesis pathway, EV content differs depending upon the cellular source and health status.

## 2. Isolation of EVs

Various methods used for EV isolation include differential ultracentrifugation, immunomagnetic-bead separation, density gradient centrifugation, chromatography, precipitation-based separation, ultrafiltration, nanoplasmon-enhanced scattering (nPES), and on-chip exosome isolation Yu LL et al. [11] 2018. The most commonly used method and the gold standard in EV isolation is differential ultracentrifugation Livshits MA et al. [12,13] 2016, Jeppesen DK et al. [12,13] 2014].

### 2.1. Differential Centrifugation—The Gold Standard

For differential centrifugation (DC), the exosome EVs are separated from macromolecular proteins, apoptotic bodies, cell debris, and cells by a series of increasing centrifugations at 4 °C culminating with ultracentrifugation at 100,000× *g* Szatanek R et al. [14] 2015. Briefly, samples are precleared by a series of low-speed spins, first 300× *g* to pellet cells, then 2000× *g* to pellet debris, and lastly, 10,000× *g* to remove the macroparticle within the supernatant. Finally, The precleared supernatant is ultracentrifuged at 100,000× *g* for 70 min. The pellet from the ultracentrifugation step results in the EV(exosomes) pellet. Given the overlap in the size of exosomes and microvesicles size, the EV pellet likely consists of both vesicle types. DC may be useful in isolating exosomal EVs but can be labor-intensive and time-consuming with a low yield. Despite these disadvantages, DC is considered the gold standard for exosome isolation.

### 2.2. Immunoaffinity

Immunoaffinity isolation of exosomes from tissue culture media or sera can be done via immunomagnetic-beads coated with monoclonal antibodies specific for tetraspanins, such as CD81, CD9, and CD63, found on the exosome surface Konadu KA et al. [15,16] 2016, Tauro BJ et al. [15,16] 2012. After the sample has been incubated with the antibody-labeled magnetic beads, the exosome-antibody-bead complexes formed are added to the separation column and retained in the column upon applying the magnetic field. This method may compromise the captured exosome integrity but assures highly purified exosomes, unlike the DC method. The primary disadvantage of immunoaffinity is the inability to separate the beads from the exosomes, thus preventing or limiting downstream applications.

### 2.3. Density Gradient—OptiPrep™

Constituents within the culture medium or serum/plasma samples are separated based on the isodensity zones formed during ultracentrifugation for density gradient-based EV isolation. The samples are layered on top of the density-gradient solution and ultracentrifuged at 10,000× g. This ultracentrifugation separates exosomes from the other sample constituents based on density and size Whiteside TL [17,18,19] 2018, Kamerkar S et al. [17,18,19] 2017, Lobb RJ et al. [17,18,19] 2015. The most commonly used solution for this method is either iodixanol (OptiPrep™ STEMCELL Technologies, Vancouver, BC, Canada) or a 30% sucrose solution. Samples layered at the top of a 30% sucrose, or iodixonal solution are ultracentrifuged, at 100,000× *g* for 16–18 h yielding exosomes at a characteristic banding density zone. This method results in exosome isolations of higher purity as a result of greater separation efficiency. However, the resulting yield, similar to DC, is low.

### 2.4. Chromatography

Chromatography can also be used to obtain exosomes uniform in size and of high purity. Particles within the sample are separated through the filtration column at differing rates via centrifugation of the column. Separation of the EV particles is based on the gel pore size and EV particle size Szatanek R et al. [14] 2015. However, this method yields a paltry exosome yield and requires high-priced specialized laboratory equipment.

### 2.5. Precipitation

Precipitation-based exosome extraction methods, such as ExoquickTM (System Biosciences) total exosome isolation (Sigma Aldrich, St. Louis, MO, USA), are commercially available. These methods are now commonly used to isolate exosomes from small volume samples Alvarez ML [20] 2012. Upon mixing the sample with the precipitating solution, a polymeric ionic web captures exosomes, which are then pelleted by centrifugation. This method grants extreme ease as it is simple to perform, quick, requires only a simple centrifuge, and provides exosomes uniform in size. However, the exosomes may be contaminated with microvesicles and proteins, impairing analysis, and potential downstream use. For the precipitation reagents, the reagent itself is expensive. Each vial usable only for a small number of samples, limiting its use due to the financial strain on research funding.

### 2.6. Ultrafiltration

Exosomes may also be separated from EVs using ultrafiltration to isolate exosomes within a sample. They pass through filters with increasingly small pore size, which traps particles of higher molecular mass and allows EV exosomes and other nanoparticles to flow through Bhattacharjee C et al. [21] 2002. This method can be accomplished using either ultracentrifugation or stirring, with the latter providing the benefit of a decreased pressure on the exosomes. Exosomes maintain their integrity during the ultrafiltration process, which is less time consuming and results in a higher exosome yield without the risk of exosomal aggregation. However, just as with the other techniques, there is likely microvesicle contamination resulting in reduced exosomal sample purity.

### 2.7. Nanoplasmon-Enhanced Scattering (nPES)

Antibodies against exosomal markers, such as CD81, are used in nPES to detect and capture exosomes, similar to an enzyme-linked immunosorbent assay (ELISA). A sensor chip with a silica surface conjugated with anti-tetraspanin antibodies captures the exosomes, which are then bound by antibody-coated gold nanoparticle probes (GNPs), forming complexes of exosomes and GNPs Rojalin T et al. [22] 2019. Exosome quantity is measured via dark-field microscopy. However, the analysis may require complex statistical tools, and exosomal protein detection may be costly. Regardless, nPES is a quick, sensitive, and high-throughput method of detecting even trace amounts of exosomes from samples.

### 2.8. Lab-On-Chip Exosome Isolation

Lab-on-chip devices, such as the exosome total isolation chip (ExoTIC), can extract exosomes via filtration, yielding purified, and enriched exosomes Liu F et al. [23] 2017. ExoTIC provides a quick, easy to use, scalable, and affordable method of generating a high yield of patient-derived exosomes, employed in downstream applications, disease diagnosis, and point-of-care testing.

Taken together, a multitude of methods are currently available for exosome isolation allowing for different degrees of purity and subsequent downstream application. As the exosome research field continues to grow, the exosome isolation techniques will also improve, allowing for development of second and third generation techniques for high yield and pure exosome isolations.

## 3. Exosomal Content and Characterization

Exosomal EVs are enriched with the protein superfamily consisting of four transmembrane domains, tetraspanins, which form tetraspanin-enriched microdomains (TEMs) impacting exosome content, EV binding and uptake by target cells, EV biogenesis, and exosome antigen presentation [11]. Tetraspanins, including CD9, CD63, and CD81, are highly enriched within the exosomal membrane and serve as excellent exosomal biomarkers [11]. TEMs assemble the proteins and facilitate the protein-protein interactions required for ILV formation, and therefore offer another ESCRT-independent mechanism of EV generation [12]. Various intracellular vesicular trafficking steps, such as trafficking and budding of vesicles, vesicle docking, and membrane fusion, are controlled by the RAB family of small GTPase proteins [13]. Endosome-associated RAB GTPases have been observed in exosomes [14]. Inhibition of either RAB35 or RAB11 impaired exosome secretion in cells bearing the proteolipid protein (PLP) exosome biomarker or the heat shock cognate (Hsc70) chaperone protein, respectively [15,16]. Additionally, the absence of RAB2B, RAB51, RAB9A, RAB27A, or RAB27B in HeLa cells, via knock-out, results in exosome secretion inhibition demonstrating that these RAB GTPases are required for exosome biogenesis [17,18]. Exosome biogenesis and autophagy have an inverse relationship [18,19]. Lastly, exosome cargo-protein, RNA, and miRNA depends on cellular status and derivation. Viral infection modulates exosome content and may play a role in the associated viral pathology [6,18,19].

## 4. Role of EVs in the Pathogenesis of Viral Infections

EV-mediated modulation of the immune system has been well studied. EVs can present antigens, trigger production and release of inflammatory cytokines, and promote cancer metastasis or induce anti-tumor responses [20,21,22,23,24,25]. There is also data indicating an interaction between EVs and viral infection. For example, EVs may facilitate viral replication and transmission by functioning as carriers of viral genetic elements, viral proteins, or regulatory elements [26]. EV biogenesis facilitates viral spread when the following conditions are met: (1) Viral proteins or RNA must reach the ILVs; components of Dengue virus (DENV), vesicular stomatitis virus (VSV), and hepatitis C virus (HCV) have all been identified in ILVs [6]. (2) Exosomes must interact with target cells releasing their infectious cargo into the extracellular space; recipient cells receive both viral and exosome constituents upon exosome entry into the cytoplasm. This condition is demonstrated by exosomes derived from HCV-infected human hepatoma cells that transport the viral envelope and core proteins alongside a full-length viral RNA [6,27]. Here we reviewed the role of EVs in the dissemination and pathogenesis of select viruses causing disease in humans.

### 4.1. Picornaviridae and Togaviridae

Hundreds to thousands of coxsackievirus, rhinovirus, or poliovirus are packaged within phosphatidylserine (PS) lipid-enriched vesicles. This packaging enables the collective transfer of multiple viral genomes to a single cell, enhancing viral replication and enabling viral quasi-species genetic cooperativity [28,29]. To spread infection, coxsackievirus B1 must induce host cell lysis [30]. However, given that EVs may carry a replication-competent viral genome or proteins, coxsackievirus B1 can spread via EVs. Evidence suggests the intercellular transmission of coxsackievirus B1 via the increased microvesicle release induces an elevation intracellular calcium concentration, resulting in depolymerization of the host’s actin cytoskeleton, a possible non-lytic cell-cell strategy to perpetuate infection [30]. Additionally, infectious virions may be transported to adjacent cells via apoptotic bodies, enhancing viral spread [3]. Pharmacologically blocking the generation of Chikungunya virus-induced apoptotic bodies in infected HeLa cells restricts viral spread to nearby cells, demonstrating the potential of hijacked apoptotic bodies in enhancing viral propagation [3].

### 4.2. Herpesviridae

EV cargo has been shown to contain several viral factors involved in viral dissemination and transmission. Exosomes transport Herpes simplex virus 1 (HSV-1) miRNA and mRNA, and potentially suppress viral reactivation to facilitate viral transmission to a new host [31]. Epstein–Barr virus (EBV), ubiquitous in humans, is a γ-herpesvirus associated with epithelial and lymphoid malignancies, a potential generator of auto-antibodies, and responsible for infectious mononucleosis [32]. Exosomal EVs derived from EBV infected B-lymphocytes release exosomes carrying MHC II molecules. Given the B-cell role in antigen presentation, these B-cell-derived EVs could activate CD4+ T-cells [6,25]. EBV-infected nasopharyngeal carcinoma cell release exosomes deliver a CD4+ T-cell apoptosis inducer and immunoregulator protein galectin-9, to evade the host immune response [6,33,34]. Natural killer (NK) cell cytotoxicity, IFN-γ production, and T-lymphocyte activation and proliferation is known to be inhibited by the latent membrane protein 1 (LMP1) of EBV; a viral oncogene commonly detected in EBV-associated tumors. Interestingly, LMP1 was found in the exosomal cargo of theses EBV-associated tumors, thereby supporting the concept that EVs play a role in facilitating viral host-immune response evasion strategy [6,33,35,36].

### 4.3. Filoviridae

Infection with the ssRNA, negative sense Ebola virus (EBOV) results in systemic infection with severe hemorrhagic fever, immune suppression or overactivation, and tissue damage [37,38,39]. Upon infection, EBOV primarily targets dendritic cells, monocytes, and macrophages, potentially facilitating systemic virus spread, including liver and secondary lymphoid organs [40]. Given the symptoms mentioned above and the high mortality rate of 80–90%, Ebola patients’ rapid identification is necessary [41]. A commonly applied technique for diagnosing Ebola patients is the detection of VP40, the EBOV matrix protein [39]. VP40 may employ two methods to release from cells, independent budding from cells or exosomal incorporation [39]. This transportation of VP40 into the nucleus facilitates EV synthesis regulation via over-transcription of cyclin D1 by binding VP40 to cyclin D1′s promoter, dysregulating the cell cycle [39]. Additionally, the VP40-laden exosomes exert a dose-dependent decrease in cellular viability of recipient monocytes and T-cells; and these exosomes contained cytokines, which may contribute to EBOV pathology [39].

EBOV pathology is further enhanced by exosome-bound VP40 modulating RNAi components, such as Dicer and Ago 1, and inducing recipient naïve cell death while upregulating exosome biogenesis [41]. EBOV content release is not limited to exosomes but extends to microvesicles as well [38]. Microvesicles containing EBOV glycoproteins (GP) have been linked to increased pathogenicity and immune evasion [38].

### 4.4. Paramyxoviridae

Respiratory syncytial virus (RSV) causes acute respiratory tract infections in the elderly, children, and immunocompromised individuals, resulting in an estimated 200,000 deaths annually [42]. To better understand RSV pathology and developing a vaccine, exosomal cargo during RSV infection was characterized [42]. Although the exosomes contain RSV components, such as the RSV nucleocapsid protein N, infectious RSV particles were undetected in exosomes, and the exosomes containing N were unable to infect cells. However, exosomes did exhibit significant changes to RNA composition that resulted in chemokine release [42]. The miRNA and piRNA content of exosomes generated from RSV infected cells was significantly modulated [42]. Some of the miRNA content was expressed at a significantly higher level within exosomes generated from RSV infected cells than uninfected cells [42]. Exosomes derived from RSV infected cells induce exposed human monocytes to secrete proinflammatory mediators, such as IP-10, RANTES, and MCP-1 [42].

Additionally, exosome-bound RNA cargo was protected from degradation, and the RNA subtype proportions were significantly modulated by RSV infection; of note, the upregulation and downregulation of miRNAs [42]. RSV infection in patients with cystic fibrosis is associated with coinfection with the opportunistic pathogen *Pseudomonas aeruginosa* [M1] [43]. The [AR2] shift from an acute *P. aeruginosa* infection to a chronic state is dependent on the formation of a biofilm with antibiotic properties, within the lung, which facilitates disease progression [43]. Infection with RSV promoted exosome-bound transferrin secretion, an iron-binding protein found in the host, known to promote *P. aeruginosa* biofilm growth [43]. This transferrin secretion demonstrates the capacity of RSV to facilitate the persistence of pathogens within the airway epithelium via exosomes [43]. However, exosomes may also facilitate the host immune response upon Influenza A virus (IAV) infection. Human tracheobronchial epithelial cells traffic components of the innate immune response, such as MUC1, MUC4, and α-2,6-linked sialic acid via exosome-like vesicles [44].

### 4.5. Orthomyxoviridae

Influenza A viruses pose a threat to humans worldwide, causing outbreaks of acute respiratory tract infections and seasonal epidemics [45,46]. About 36,000 individuals die as a result of flu-associated infections annually in the US [45]. Intercellular communication via exosomal miRNAs may modulate cell function, alter recipient cell pathways, and facilitate viral persistence [45,46,47,48]. IAVs have been found to alter circulating miRNAs within exosomes, potentially promoting viral pathogenesis [49,50]. For example, IAV-infected human lung adenocarcinoma epithelial A549 cells produced exosomes containing miRNA hsa-miR-1975, which inhibited IAV replication by inducing interferon production [51]. IAV modulation of exosomal cargo is not limited to miRNAs, as autophagy-related proteins, including Atg3/7 and antiviral cytokines such as IL6, IL18, and TNF, are found in exosomes released from IAV-infected macrophages [52]. This finding demonstrates IAV capacity to alter macrophage-dependent innate immune responses and intercellular cell signaling via manipulation of exosomal cargo. Pathogens may also transport viral components within exosomes, such as transportation of IAV progeny RNA to the apical side of the membrane by attaching to Rab11 vesicles, thereby facilitating late-stage IAV budding and infection [6,53]. Applying LC-MS/MS in proteomic studies have discovered that IAVs integrate exosomal proteins or markers such as annexin A3, CD9, CD81, and ICAM1, contributing to the influenza virion structure, viral spread, and implying a shared formation pathway with exosomes [54].

### 4.6. Hepadnaviridae

Hepatitis B virus (HBV) infects human hepatocytes leading to liver fibrosis, cirrhosis, and eventually hepatocellular carcinoma [55,56]. HBV’s HBx protein facilitates oncogenic activities via various mechanisms such as host gene stimulation, cell cycle interference, and mitogenic signaling [55]. Both HBx protein and associated mRNA encapsulated within exosomes released from HBV infected cells into the extracellular environment, permitting horizontal transfer of its gene products and viral protein expression [55]. HBx containing exosomes have significantly different cargo, both quantitatively and qualitatively [55]. These altered exosomes promote HBV-associated liver diseases by inducing proliferative signaling and enhancing exosome biogenesis via increasing neutral sphingomyelinase 2 activity [55]. The natural killer group 2D (NKG2D) receptor recognizes ligands on infected cells, promoting innate immunity and lymphocyte activation to defend the host from infections [57]. Exosomes generated from HBV infected cells contain viral RNA that induces expression of the NKG2D ligand in macrophages, implying a role for HBV-infected cell-derived exosomes in NK cell activation. The upregulation of CD69 confirms the exosome role in modulating NK cell activation and inducing IFN-γ production, which leads to the degradation of viral RNA in hepatocytes [56].

Furthermore, infection with HBV increased immunosuppressive miRNAs: miR-21 and miR-29a, within CD81+ exosomes and EVs, transferred from hepatocytes to macrophages [56]. Downregulation of IL-12p35 and IL-12p40 occurs as a result of miR-21 and miR-29a expression, respectively. The increase in these miRNAs potentially inhibit NK cell activity via IL-12 downregulation and facilitating viral evasion of the host immune response [56]. Another study concluded through proteomic analysis of exosomes via LC-MS/MS that HBV-infected HepAD38 hepatoblastoma cell line-derived exosomes contain HBV-associated proteins capable of significantly reducing monocyte IL-6 production [58]. HBV infection in HepAD38 cells alters 35 exosome-bound proteins, including the increase of five proteasome subunit proteins: PSMD1, PSMD7, PSMD14, PSMC1, and PSMC2, enhancing proteolytic activity [58]. Inhibition of exosome-dependent proteasomal activity resulted in increased IL-6 production, implying proinflammatory molecules modulated by proteasomal subunit proteins within HepAD38 exosome transport [58]. Exosomes transfer HBV proteins and genetic content to other cells. For example, NK cells and hepatocytes of chronic HBV patients release exosomes that contain HBV proteins and nucleic acid [59]. Uptake of these exosomes impairs NK cell production of IFN-γ, NK cell survival and proliferation, cytolytic activity, and NK cell responsiveness to poly (I:C) stimulation [59]. The exosome role in HBV infection is not limited to facilitating viral replication. IFN-α induced HBV antiviral activity is transferred via exosomes from liver non-parenchymal cells (LNPCs) to hepatocytes [60].

### 4.7. Flaviviridae

Currently, there is no vaccine against HCV, a +ssRNA flavivirus that is one of the leading causes of liver disease worldwide [61]. Sera from HCV-infected patients and supernatants of J6/JFH1, an HCV-infected Huh7.5 cell line, contain exosomes with HCV RNA, proteins, and particles [61]. Viral genome packaging in exosome is not limited to the Picornaviridae, HCV-infected hepatocytes release full-length genomic HCV RNA laden exosomes that can activate immune cells and establish a productive infection in naïve human hepatoma cells to facilitate viral spread [62]. Human Ago2 and miR-122, necessary for HCV RNA accumulation and translation, have been detected within exosomes derived from HCV-infected patient serum or J6/JFH1-HCV-infected Huh7.5 cells. This demonstrated the exosome capacity to enhance viral spread via transport of viral regulatory elements [61,63]. EVs are also capable of transmitting Flaviviruses from arthropod vectors to humans by acting as carriers [18].

Arthropod-borne neurotropic encephalitis viruses replicate within the peripheral tissues and blood of a vertebrate host after transmission, cross the blood-brain barrier (BBB), and infect the central nervous system (CNS) [64]. Langat virus (LGTV)-infected *Ixodes scapularis* ISE6 tick cells release EVs, which mediate the transmission of viral RNA, envelope protein, and non-structural 1 (NS1) protein from arthropod to human cells [64]. These insect-cell derived exosomes are also delivered upon infection of naïve human skin keratinocytes (HaCaT cells), the barrier that first contacted the tick bites [64]. LGTV infects murine brain endothelial barrier (bEnd.3) cells, and the endothelial cells produce exosomes, which transmit infectious RNA and proteins to murine neuronal (N2a) cells [64]. LGTV infected neuronal cells further disseminate the virus within the brain via exosomes resulting in neuronal loss and neuropathogenesis [64].

#### 4.7.1. ZIKA

To date, few studies have demonstrated that exosomes are integral to the interaction between ZIKV and host cells. ZIKV and Zika viral proteins have been detected in the eyes and semen, months after initial infection, thus creating a need to comprehend how these particles persist and damage neuronal cells. Exosomes can easily cross the endothelial barriers protecting these sites and therefore are a potential antigen source [65]. Additionally, precedence for modulating EV contents by ZIKV exists, as other flaviviruses have been observed to do so [65]. Here this review summarized the role of EVs in the context of ZIKV infection.

ZIKV has been demonstrated to cross the placental barrier (PB), detecting ZIKV in the fetal brain and amniotic fluid confirming ZIKV tropism for neuronal tissue [66,67,68]. Infection of the fetal brain with ZIKV may result in severe congenital malformations, also known as ZIKV fetal syndrome [66,67,68]. Individuals with ZIKV fetal syndrome may present with several congenital disabilities, including but not limited to: facial disproportionality, microcephaly, hypertonia, cutis gyrata, ventriculomegaly, and a lack of brain tissue [68]. It is believed that after crossing the placenta, ZIKV damages neuronal cells and induces the immune response [68]. Although the exact mechanism for ZIKV passage through placental trophoblasts is unknown, ZIKV, like DENV, may employ the placental exosome pathway at the trophoblast ER for this purpose, as it is strongly associated with the process of secretory autophagy [66]. Immature ZIKV viral particles translocate to the trans-Golgi network, from the ER [67]. ZIKV may be vertically transmitted independently of the secretory autophagy pathway due to increased permeability resulting from ZIKV-induced damages and apoptosis of placental cells [66]. Studies indicate a role for autophagy in ZIKV-associated neuropathology, as inhibition of autophagy results in inhibition of ZIKV replication [68].

Uninfected human placental trophoblast (HPT) cells secrete type-III IFNs, IFNλ1 and IFNλ2, conferring anti-ZIKV protection [69]. Syncytiotrophoblasts constitutively generate IFNλ1, providing an antiviral state protecting placental cells in an autocrine manner and non-placental cells in a paracrine manner [69]. Non-placental cells may be protected and establish an antiviral state via exposure to HPT-derived conditioned media (CM) [69]. This data suggests that ZIKV replication within placental syncytiotrophoblasts permitting access to the fetal compartment is not possible without either ZIKV evasion of the IFN type III antiviral properties or bypassing the PB via an unknown pathway, possibly EV-mediated [69].

#### 4.7.2. EV-Mediated Restriction of ZIKV Pathogenesis

Exosomes and microvesicles are released into maternal blood during pregnancy. They can be recovered during the first and second trimester of pregnancy, increasing in concentration with the progression of the pregnancy [70]. These exosomes have been found to contain primate-specific chromosome 19 cluster (C19MC) miRNAs functioning as antiviral agents, which may be transferred to non-placental cells, conferring protection and upregulating autophagy upon the target cells if delivered via exosomes generated from trophoblasts [70,71]. EVs carrying MIR517-3p, MIR16B-5p, and MIR512-3p induced potent antiviral activity in recipient cells [70]. Additionally, HPT-derived exosomes carry miRNAs conferring viral resistance to non-placental recipient cells [71]. The miRNAs of C19MC attenuated ZIKV infection in non-HPT cells, however, they fail to activate IFN-stimulated genes [72]. Together, this data demonstrates the potential anti-ZIKV properties of HPT-derived exosomes, acting in an IFN-independent manner.

ZIKV infection of astrocytes significantly increases EV biogenesis, predominantly composed of microvesicles and exosomes [73]. Additionally, significant variation of miRNA transcripts expression has been observed in HPTs following permissive replication of ZIKV. For example, downregulation of miR-21, known to cause TLR7-mediated neurotoxicity [74,75]. This data presents with an apparent anti-ZIKV host cell response transported via exosome-trafficking.

Inhibition of ZIKV infection by EVs derived from the semen of a ZIKV-infected patient has been observed [76]. Although freshly derived ZIKV-infected patient semen efficiently blocked ZIKV-MR766 infection of Vero E6 cells, the nature of the antiviral component responsible for this inhibition remains unknown [76].

#### 4.7.3. EV-Mediated Enhancement of ZIKV Neuropathology

Upon infection with ZIKV, macrophages, which are permissive to ZIKV infection, are recruited and amplify ZIKV replication [77]. The exosomes generated from the activated macrophages are transported to the human placenta, leading to the induction of placental pro-inflammatory cytokine production [78]. In combination with ZIKV NS5-mediated activation of NLRP3, inducing stimulation of human macrophage IL-1β secretion, which results in the host inflammatory response, macrophage recruitment promotes inflammation, a major determinant of ZIKV pathogenicity [79].

**Figure 3:**
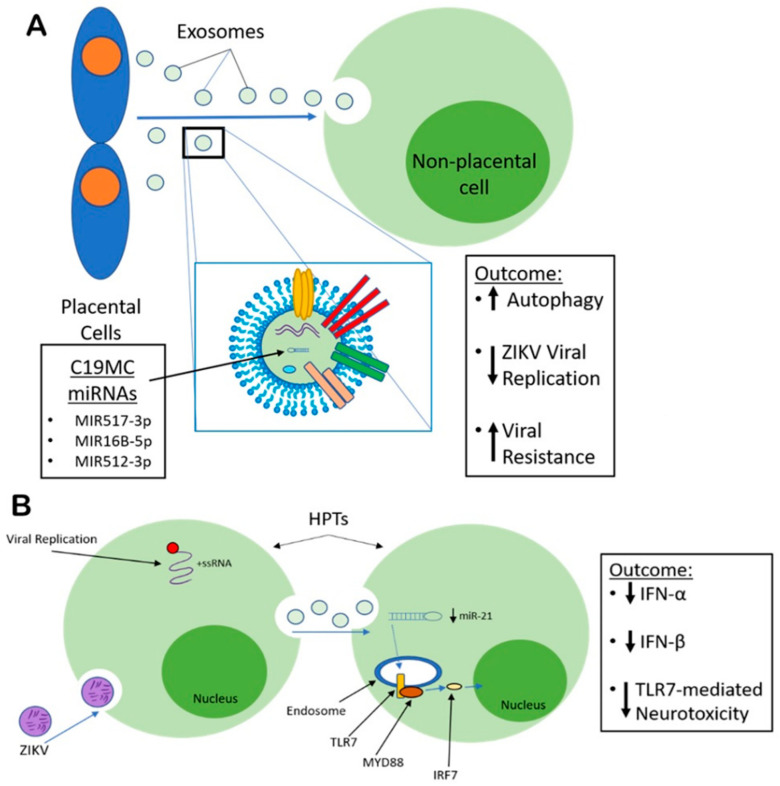
EV-mediated anti-ZIKV effects. (**A**) HPT cell-derived EVs bound with miRNAs with potent anti-viral properties, have been detected. The EVs migrate to non-placental recipient cells conferring anti-ZIKV protection and up-regulating autophagy. (**B**) ZIKV infection of HPT cells results in an anti-ZIKV host-cell response transported via exosome-trafficking, downregulating miR-21 in uninfected HPTs, reducing TLR7-mediated Neurotoxicity.

A murine study has shown that exosomes facilitate the transmission of ZIKV across neurons by functioning as mediators [80]. An increase in exosome biogenesis was recorded in mouse cortical neuronal cell-derived exosomes alongside the detection of ZIKV-RNA and envelope (E) protein [80]. Furthermore, neutral sphingomyelinase (nSMase)-2/SMPD3 gene expression and activity was induced by ZIKV [80]. SMPD3 regulates exosome generation and release [80]. Exosome-mediated viral transmission rate and burden were reduced after the silencing of SMPD3 in neuronal cells; a similar effect occurred upon exposure to GW4869, an inhibitor specific to SMPD3 [80]. This study suggests that modulation of SMPD3 activity resulting from ZIKV cortical neuron infection is integral towards viral infection and exosome-mediated transfer resulting in ZIKV-associated neuropathology, such as microcephaly, as a result of severe neuronal death [80]. At this time, there are not many clinically relevant interactions between EVs and ZIKV are known; research is ongoing (Figure 3). Unlike ZIKV, however, a greater volume of EV-mediated effects on HIV-1 pathogenesis has been studied.

### 4.8. Retroviridae

#### 4.8.1. Human Immunodeficiency Virus Type 1 (HIV-1)

HIV-1 targets both macrophages and T-lymphocytes via the primary receptor CD4 and coreceptors CCR5 or CXCR4, thus inducing apoptosis of CD4+ receptor T-lymphocytes and weakening the host immune system [81,82]. With over 75 million infected individuals worldwide, HIV remains an epidemic and one of the most significant causes of morbidity and mortality [82]. Even though antiretroviral therapy (ART) successfully restricts HIV-1 infection, often reducing viral loads below detection, challenges continue [18,82]. Retroviruses are unique in that they have both lytic and latent life cycles. ART is not curative due to either the failure to eliminate latent virus reservoirs, treatment toxicity, viral mutations, or divergent patient responses to HIV infection, therapies, or adherence [18,82]. Therefore, comprehending the HIV-1 interaction with immune cells is integral to elucidate novel aspects of HIV-1 disease, which could be developed as therapeutic targets [18,82].

HIV-1 particles and exosomes share some molecular properties of biogenesis, and cellular uptake mechanisms, all of which are beyond this review [18]. There are critical points in the viral assembly that intersect EV biogenesis; therefore, exosomal EVs play a crucial role in HIV-1 pathogenesis [83]. This indicates potential roles for exosomes in HIV-1 pathogenesis, which is covered here [18].

#### 4.8.2. EV Interaction with Host Cell Restriction Factors and HIV

EVs contribute to antiviral responses by delivering host-derived restriction factors to nearby cells. For example, the host cell viral restriction factor cellular cytidine deaminase APOBEC3G (A3G) is contained within CD4+ T-cell derived EVs. A3G inhibits HIV-1 replication by interfering with HIV-1 reverse transcription in a deamination-dependent and deamination-independent manner, catalyzing hypermutation of the viral DNA [84,85,86]. However, this effect is not observed in vivo, as A3G is depleted post-HIV-1 infection, leaving an insignificant quantity of EV-bound A3G incapable of having an antiretroviral impact [18,86].

CD4+ T-lymphocytes release exosomes with CD4 on the surface, thereby competing with cells for binding to HIV-1 virions. The CD4+ EVs can potentially restrict HIV in several ways: acting as a decoy for CD4+ T-cells, neutralizing HIV-1 virions, or protecting neighboring T-cells from infection, ultimately inhibiting HIV-1 spread. These effects of CD4+ exosomes can be countered by the HIV-1 accessory protein, Nef, which reduces CD4 expression in T-cells [18,87]. CD4+ EV effects suggest that CD4+ T-cells utilize exosomes to protect against HIV-1 infection, indicating a role for EVs in antiviral immunity.

#### 4.8.3. Immune Cell-Derived EVs and Antiviral Effects

Similar to CD4+ T-cells, CD8+ T-cells release exosomes that restrict HIV-1 replication. CD8+ T-cell-derived EVs contain an anti-HIV protein moiety that suppresses replication without EV internalization. This indicates that exosome-mediated HIV-1 transcription suppression may comprise of an intracellular signaling pathway [88]. EVs contain components of toll-like receptor (TLR) innate antiviral pathways. TLR3-activated human brain microvascular endothelial cells (HBMEC) release EVs that block HIV-1 infection to the CNS via transport of antiviral factors and IFN-stimulated genes (ISGs), thereby transferring anti-HIV protection to macrophages [89]. Macrophage and CD4+ T-cells, enriched in the gastrointestinal system (GI), are protected against HIV-1 by EVs released from TLR3-activated intestinal epithelial cells (IECs) containing HIV-restriction miRNAs (miRNA-20 and miRNA125b) and ISGs (ISG15, OAS-1, and Viperin) [90]. These mechanisms demonstrate that EVs interact with cellular barriers (BBB/GI) via TLR pathways to promote innate antiviral immunity [89,90].

EVs are found within most bodily fluids, including blood, breast milk, semen, and vaginal fluids, hindering HIV-1 infection throughout the body [91,92,93,94,95]. Vertical transmission of HIV-1 can be inhibited by breast milk-derived EVs that bind DC-SIGN receptors, thereby preventing HIV-1 from binding and potentially inhibiting HIV interaction with monocyte-derived dendritic cells (MDDCs) and HIV-1 transfer to CD4+ T-cells [94]. Sexual transmission of HIV-1 is inhibited by EVs derived from uninfected semen or vaginal fluid-derived EVs, negatively affecting reverse transcriptase activity [92,93]. Heterosexual transmission of HIV-1 is facilitated by vaginal epithelial cells (VECs) uptake of semen-derived EVs containing functional viral mRNA, enabling viral spread, as evidenced in a murine AIDS model [92]. However, in a human transwell model using VECs, semen-derived EVs inhibited HIV-1 spread [92]. EVs play a dichotomous role in modulating HIV-1 transmission, restricting and enhancing viral spread [93,94].

#### 4.8.4. EV-Mediated Enhancement of HIV-1 Infection

EVs also enhance HIV-1 pathogenesis and infection [18]. HIV-1 infection/pathogenesis disrupts the endomembrane system and modulates EV cargo, biogenesis, targeting, and/or release frequency [18]. For example, PBMCs release microvesicular EVs containing CCR5, transporting them to neighboring cells deficient in CCR5, enhancing cellular susceptibility to HIV-1 [96]. Megakaryocytes release EVs containing CXCR4, delivering the HIV-1 coreceptor to nearby tissues lacking CXCR4 expression, facilitating viral spread [97,98].

EVs also assist in HIV-1 entry by interacting with HIV-1 virions, which contain high concentrations of phosphatidylserine (PS), a hallmark of apoptosis. PS interacts with T cell immunoglobulin and mucin proteins (TIM-4), highly expressed in EVs. TIM-4 binding to HIV-1 PS surface-bound moieties results in increased exosome-mediated trafficking of HIV-1 to immune cells [99,100]. HIV-1 entry into human T-cell and monocytic cell lines can be enhanced by exosomal tetraspanins CD9 and CD81 [99,100]. Blocking of TIM-4 or any of these tetraspanins with antibodies, result in significant blockage of viral entry, supporting an exosome-dependent mechanism for HIV-1 entry [99,100]. TIM-4 has also functioned as an apoptotic cell phagocytic receptor due to recognizing the exposed PS moieties [101,102]. HIV-1 can evade immune surveillance by camouflaging itself within exosome aggregates, facilitating viral spread [103].

Additionally, exosomes derived from HIV-1 infected primary cells are abundant with transactivating response (TAR) element RNA, which has enhanced undifferentiated naïve cell susceptibility to HIV-1 infection [104]. Primary macrophage exposure to these HIV-1 infected cell-derived exosomes promotes macrophage release of proinflammatory cytokines, TNF-β, and IL-6, indicating EV-mediated modulation of proinflammatory cytokine gene expression [104]. EV-mediated enhancement of HIV-1 infection is not restricted to exosomes, as apoptotic bodies generated during HIV infection inhibit the function of dendritic cells (DC), resulting in decreased DC-dependent cytokine production and T/NK-cell priming via DC-CD44 receptor binding of apoptotic microvesicles [3]. Taken together, HIV–EV interactions exploit apoptotic clearance mechanisms and facilitate viral replication and host cell viral uptake [18,101,102,105].

Besides facilitating viral entry, EVs transfer active HIV-1-derived molecules to bystander cells, promoting viral infection [106]. HIV-1 infected cells release EVs containing the HIV-1 envelope (Env) protein (gp120), Gag, and Nef. EVs containing gp120 significantly raise HIV-1 infectivity in lymphoid tissues [106,107,108,109]. Nef, an accessory protein responsible for modulating protein trafficking within host cells and HIV-1 pathogenicity, is known to be released within EVs [110,111,112]. EVs-containing Nef has been identified at high concentrations in the plasma of HIV-1 infected patients. Nef-containing EVs function to promote EV secretion, increased MVBs within cells, and induce apoptosis within CD4+ T-cells [110,111,112,113]. Given that Nef has several functions, Nef-EVs could potentially promote decay of CD4+ T-cell populations, promote CD8+ T-cell activity, CXCR4-mediated apoptosis, and ADAM17 activation, increasing CD4+ T-cell permissiveness to HIV-1 [114,115,116,117,118].

Viral components enhancing HIV-1 infection are not limited to viral proteins. Through coevolution with the host, HIV-1 presents with differential miRNA content relative to uninfected cells, facilitating suppression of host RNA interference (RNAi) [119,120]. EVs from HIV-1 infected macrophages and plasma contain HIV-1-derived miRNAs: vmiR88 and vmiR99, promoting macrophage release of TNF-α via TLR8 activation, thus supporting chronic immune activation [121]. Exosomal and cellular miRNA profiles are modulated by the HIV-1 Nef protein, affecting HIV-1 pathogenesis and viral replication modulated by host RNAi [122]. EV-encapsulated miRNAs can alter HIV-1 pathology and enhance an infection similar to proteins.

**Figure 4:**
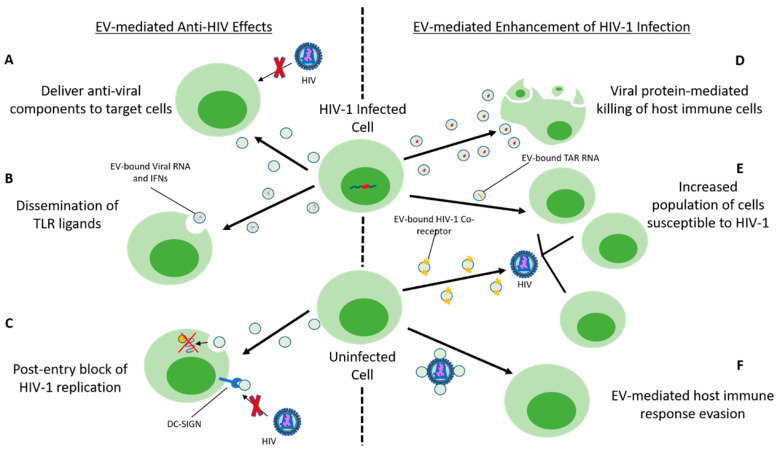
HIV-1 infected cell-derived EV-mediated anti-viral and pro-viral effects. Upon infection with HIV-1, cells release EVs which may modulate HIV-1 pathogenesis, either restricting infection or enhancing it. (**A**) EVs can deliver anti-viral particles, such as A3G, inhibiting HIV-1 replication. (**B**) EV-mediate dissemination of TLR ligands, including HIV-restriction miRNAs, ISGs, IFNs, and anti-viral factors transfer anti-HIV protection and alert neighboring cells of ongoing infection. (**C**) EVs derived from bodily fluids such as breast milk, semen, and vaginal fluids can hinder HIV-1 infection by blocking HIV-1 replication after viral entry or competing with HIV-1 for receptor access. Breast milk-derived EVs compete with HIV-1 binding to the DC-SIGN receptor, preventing vertical transmission. Internalization of either semen or vaginal fluid-derived EVs results in deleterious effects upon HIV-1 reverse transcriptase activity leading to a post-entry block of HIV-1 replication. (**D**) EV-mediated transport of viral particles, such as HIV-1 Nef protein, triggers viral-mediated apoptosis of anti-viral immune cells. (**E**) Transport of HIV-1 chemokine co-receptors CCR5 or CXCR4 via EVs to cells deficient in these receptors, generates new populations of cells which are now susceptible to HIV-1 infection. HIV-1 infected primary cell-derived EVs carry TAR element RNA, enhancing susceptibility to HIV-1 infection in undifferentiated naïve cells. (**F**) Lastly, EVs may aggregate upon the HIV-1 virion as a result of exploitation of exosomal surface properties, camouflaging HIV-1 and facilitating viral spread to uninfected innate and adaptive immune cells.

HIV-1 crosses the BBB, enters the CNS, infecting astrocytes, and microglia, causing chronic neurologic disease [123]. BBB permeability and integrity are disrupted by microglia-derived Nef-EVs, which reduce zonula occludin-1 (ZO-1), lower tight junction (TJ) protein expression in HBMECs, and increase TLR-induced chemokines and cytokines [124]. This loss of BBB integrity would induce some degree of neuropathology. Nef-EVs are elevated in the plasma of individuals with HIV-1-associated neurocognitive disorders (HAND), thus suggesting a role of exosome-encapsulated Nef in HIV-1 neuropathology [110]. Overall, EVs enhance and restrict HIV-1 infection through various methods, summarized in (Figure 4).

### 4.9. Coronaviridae

Coronaviruses belong to a family of enveloped positive-strand RNA viruses encoding a standard set of four structural proteins: the small envelope glycoprotein (E), membrane protein (M), nucleocapsid protein (N), and the spike glycoprotein (S) [125,126,127]. Coronaviruses cause disease in humans and animals. There are four human coronaviruses 229E, NL63, OC43, and HKU1 that cause a mild illness similar to the flu with temperate systems. However, three pathogenic strains of coronaviruses cause an atypical pulmonary disease, known as severe acute respiratory syndrome (SARS), in humans SARS CoV, Middle Eastern respiratory syndrome (MERS-CoV), and now the novel SARS CoV-2 of 2019. The first SARS coronavirus outbreak with SARS CoV in 2002 has been contained primarily in China since 2003. The most recent coronavirus outbreak in 2019 is with SARS CoV-2, associated with the severe respiratory disease called Coronavirus Disease 2019 (COVID-19) and has spread beyond China’s borders, becoming a public health issue and a pandemic in which many lives have been lost [125].

Exosomes transport the viral genome of SARS-CoV-2 to target cells [128]. Uptake of these SARS-CoV-2 exosomes by human-induced pluripotent stem cell-derived cardiomyocytes (hiPSC-CMs) resulted in an upregulation of genes associated with inflammation in hiPSC-CM [128]. Additionally, viral RNA fragments were detected within the hiPSC-CMs after coincubation with the SARS-CoV-2 gene overexpressing A549-derived EVs [128]. This exhibits how coronaviruses may infect target cardiomyocytes indirectly, without requiring a direct viral infection, instead of utilizing exosomes to deliver viral RNA. This is an excellent example of how coronaviruses may exacerbate pathology by altering the inflammatory state via exosomes.

Human host factor angiotensin-converting enzyme 2 (ACE2) has been identified, during the first coronavirus epidemic, as the receptor for the SARS causing coronavirus [129]. ACE2 and the DC-SIGN family of receptors bind the S protein, mediating coronavirus entry to target cells [125]. Given this most recent outbreak of coronavirus, there is an urgent need for a vaccine against this virus [125]. Given the physiological properties of exosomes, researchers have investigated the potential of exosomal vaccines [125]. Exosomes incorporated with spike S proteins results in the generation of neutralizing antibodies.

Furthermore, priming with the S-protein exosome vaccine and subsequent boosting via the addition of the adenoviral vector vaccine yielded neutralizing antibody titers exceeding those of a SARS-convalescent patient serum [125,130]. This exosome-based vaccine is an excellent example demonstrating the possibilities of exosomes as nanotherapeutics. Vaccines are not the only possibility as already clinical trials using exosomes derived from allogeneic bone marrow mesenchymal stem cells (ExoFlo) have begun [131]. ExoFlo was used to treat 24 patients, testing positive for SARS-CoV-2 via polymerase chain reaction [131]. Patients were classified with severe COVID-19 and moderate to severe acute respiratory distress syndrome (ARDS) [131]. A single dose was administered intravenously, with the 15 mL ExoFlo treatment being evaluated daily for 14 days [131]. Treatment with ExoFlo caused patients’ oxygenation and clinical status to improve. These improvements extended to improved neutrophil counts, with a mean reduction of 32%, and increased CD3, CD4, and CD8+ lymphocyte levels, improving patients’ lymphopenia [131]. ExoFlo is a top candidate for an exosome-based nanotherapeutic for COVID19, given its overall safety, capacity to restore oxygenation, downregulate the cytokine storm, and reconstitute immune function. [131].

### 4.10. Polyomaviridae

Polyomaviruses possess icosahedral symmetry, are non-enveloped, are small at 44 nm in diameter, are prevalent throughout nature, are composed of 72 capsomers, and adapt to thrive in the specific tissue and species they infect [132]. There are five polyomaviruses that infect humans: Merkel cell, JC, BK, KI, and WU polyomavirus [132]. Interaction between the virion and cell surface sialic acids is responsible for virion adsorption to the cell surface and subsequent cellular infection [132]. The host cell engulfs the polyomavirus via endocytosis, allowing it to enter the cell cytoplasm and, via the cytoskeleton transport machinery, transporting the virions to the nucleus [132]. Once at the nucleus, viral DNA replication and virion progeny formation occurs [132]. The release of new virions is believed to occur via either secretion from the plasma membrane or the lysis of host cells [132].

The endemic JC polyomavirus (JCPyV) is observed to have established a persistent infection in the urogenital system of over 50–70% of the human population worldwide [133,134]. In immunocompromised patients, JCPyV spreads to the central nervous system, infecting oligodendrocytes, and results in rapid progression to the severely debilitating demyelinating disease: progressive multifocal leukoencephalopathy (PML) [133]. JCPyV was found to associate with EVs in addition to infecting target cells in a receptor-independent manner [133]. Viral particles have been observed to be packaged within EVs and attached to the vesicle’s outer side [133]. Additionally, when cells were treated with neuraminidase, an enzyme that destroys receptors, viral infection was inhibited. However, neuraminidase treatment could not inhibit EV-associated viral infection [133]. Furthermore, cells failed to be transduced with mutant pseudoviruses, if possessing defective sialic acid receptor binding. However, when associated with EVs, cellular transduction became possible [133]. This EV-based mechanism of infection may play a crucial role in the spread and dissemination of JCPyV to and within the central nervous system [133].

In another study, JCPyV was observed to infect choroid plexus epithelial cells and then found to be contained within the EVs derived from these infected choroid plexus epithelial cells, trafficking the virus from the periphery to the brain, and subsequently transmitting the infection to human glial cells [134]. These JCPyV-containing EVs may be taken into the target glial cells via two methods: micropinocytosis or clathrin dependent endocytosis [134]. According to this data, infection of the parenchyma is crucial to viral spread to the CNS, demonstrating the potential of EVs as carriers of virions [134]. A study by Giovannelli et al. demonstrated that JCPyV-infected cell derived exosomes, carrying viral miRNAs, may be trafficked to uninfected cells [135].

Most individuals are asymptomatics carriers of BK polyomavirus (BKPyV), which is responsible for nephropathies in recipients of kidney transplants [136]. Handala et al. confirmed, via electron microscopy, that a single EV originating from an infected cell could transport dozens of viral particles [136]. Additionally, the EV-associated BKPyV employed a cellular entry pathway differring from that of a non-EV-associated virion, as the EV-associated viral particles did not attach to the cell via cell surface sialylated glycans nor were they able to agglutinate red blood cells [136]. Overall, current research suggests that exosomes provide polyomaviruses a significant increase in tropism and virulence. However, there is a lack of data describing the mechanism for polyomavirus’ immune evasion and persistence [136].

## 5. Therapeutic Potential of EVs as Antiviral Agents

Exosomes are ideal therapeutic agents, as they are non-immunogenic, can pass cellular barriers, and the contents can be manipulated. Exosomes can be used as delivery systems to transfer pharmaceutical drugs, proteins, enzymes, antibodies, and other biologically relevant molecules to target cells [137,138,139]. Since exosomes possess a unique biologic potential for biomedical applications given their <100 nm nanoscale size, these EVs have become attractive nanostructures to treat a viral infection or their associated neuropathologies [137,138,139].

Potential treatments against viral infections are currently being investigated and range from vaccines to therapeutic drugs [140,141]. Antiviral agents must be carried across cellular barriers, such as the placental barrier (PB) or the BBB, to reach target cells [141]. The development of EVs as nanocarriers to transport therapeutic agents across the PB may provide a drug/gene delivery system capable of treating a ZIKV infection in-utero or delivering therapeutics across the BBB to treat a neurotropic virus infection of the CNS.

EVs are capable of transporting both hydrophobic and hydrophilic molecules. Hydrophilic compounds are stored in the interior, and hydrophobic agents embedded within the lipid membrane. However, unlike liposomes, exosomes are not optimized for the encapsulation of hydrophilic macromolecules [138]. Thus, liposomes can carry both hydrophobic and hydrophilic drugs and molecules to a target site, whereas exosomes will face challenges in the encapsulation of hydrophilic agents [138]. Additionally, the loading capacity of the exosomes is low due to the presence of proteins and nucleic acids already within [138]. To improve cell targeting, exosomes may be generated by cells expressing ligands with a high binding affinity to the target cells. However, for liposomes, using a functionalized polymer to coat the liposome, generating a nanobin that improves targeted drug delivery [138]. For neurotropic viruses, EVs could deliver therapeutics across the BBB, potentially reducing viral-associated neuropathology [124,142]. Inhibition of the ESCRT machinery is a promising option for HIV-1 therapeutics since HIV-1 appropriates ESCRT. TSG101 disruption via the small molecule inhibitor FGI-104 could prevent HIV-1 pathogenesis [143]. More research is needed to elucidate both the mechanism of action and potential side effects of FGI-104. Overall, given EV properties, EVs may serve as the next class of antiviral therapeutics, with fewer side effects and outstanding biocompatibility.

## 6. Conclusions

EVs play dichotomous roles in viral infections and pathology. In this review, we summarize clinically relevant viruses shown to interact with EVs and EV-mediated effects on viral infection and pathology, summarized in (Table 1). Not only are EVs critical for intracellular communication, but they may also represent a novel innate antiviral mechanism. Viruses exploit the EV biogenesis pathway to promote: viral infection, replication, spread, and pathology. Aside from promoting their pathology, viruses use EVs to modulate antiviral immune responses.

## Figures and Tables

**Table 1:** Summary of EV-mediated effect in viral pathogenesis.

**Patdogenesis**	**Effect on Patdogenesis**	**Virus**	**Component**	**Outcome**	**Reference**
**Picornaviridae and Togaviridae**					
Viral packaging within vesicles.	Enhancement	Picornaviridae	Phosphatidylserine (PS) lipid-enriched vesicles	Increased viral replication	[41,42]
Depolymerization of the host’s actin cytoskeleton	Enhancement	Coxsackievirus B1	Increased intracellular calcium concentration	Increased non-lytic viral spread	[43]
Increased EV biogenesis	Enhancement	Coxsackievirus B1	Replication competent genome within EVs	Increased viral spread	[43]
Infectious virions hijacking apoptotic bodies	Enhancement	Chikungunya virus	Apoptotic bodies	Increased viral spread	[3]
**Herpesviridae**					
miRNA and mRNA are transported via exosomes	Enhancement	HSV-1	Exosome-bound miRNA and mRNA	Suppressed viral reactivation, facilitating viral transmission to new host	[44]
EV-bound MHC-II activates CD4+ T-cells	Inhibition	EBV	EVs derived from EBV infected B-lymphocytes	Potentially activate CD4+ T- lymphocytes	[6,38]
Transport of immunoregulator protein galectin-9, a CD4+ T-cell apoptosis inducer	Enhancement	EBV	EBV-infected nasopharyngeal carcinoma cell-derived exosome-bound immunoregulator protein galectin-9	Increased evasion of host immune response	[6,46,47]
Inhibition of Natural Killer (NK) cell cytotoxicity, IFN-γ production, and T-lymphocyte activation and proliferation by LMP1	Enhancement	EBV	EV-bound LMP1	Increased evasion of host immune response	[6,46,48,49]
**Filoviridae**					
Transportation of VP40 into the cell nucleus and subsequent binding of VP40 to cyclin D1′s promoter	Enhancement	EBOV	VP40-laden exosomes	Facilitates the regulation of EV synthesis via over-transcription of cyclin D1, dysregulating the cell cycle.	[52]
Transportation of VP40 into the cell nucleus	Enhancement	EBOV	VP40-laden exosomes	Exerts a dose-dependent decrease in cellular viability of recipient monocytes and T-cells	[52]
Modulation of RNAi machinery, such as Dicer and Ago 1	Enhancement	EBOV	VP40-laden exosomes	Inducing cell death of recipient naïve cells while upregulating exosome biogenesis.	[54]
**Paramyxoviridae**					
RSV infection upregulated expression of select exosome-bound miRNA and piRNA content	Enhancement	RSV	Exosomes generated from RSV infected A549 cells	Increased exosomal miRNA and piRNA content	[55]
Exposure of PBMC-isolated human monocytes to exosomes derived from RSV infected cells	Enhancement	RSV	Exosomes generated from RSV infected A549 cells	Induced the secretion of proinflammatory mediators, such as IP-10, RANTES, and MCP-1	[55]
**Orthomyxoviridae**					
Intercellular communication via exosomal miRNAs	Enhancement	IAVs	Exosomes generated from IAV infected cells	Modulate cell function, alter recipient cell pathways, facilitate viral persistence, and alter circulating miRNAs	[58,59,60,61,62,63]
Exosomes containing miRNA hsa-miR-1975	Enhancement	IAVs	IAV-infected human lung adenocarcinoma epithelial A549 cell-derived exosomes	inhibit IAV replication by inducing interferon production	[64]
The transportation to the apical side of the membrane of IAV progeny RNA by attaching to Rab11 vesicles	Enhancement	IAVs	Exosomes generated from IAV infected cells	Facilitating late stage IAV budding and infection	[6,66]
IAVs integrate exosomal proteins or markers such as Annexin A3, CD9, CD81, and ICAM1	Enhancement	IAVs	Exosomes generated from IAV infected cells	Contribution to the influenza virion structure, viral spread	[67]
**Hepnaviridae**					
HBV HBx protein-mediated host gene stimulation, cell cycle interference, and mitogenic signaling	Enhancement	HBV	HBx protein and mRNA encapsulated within exosomes	Permits horizontal transfer of its gene products, expression of viral protein, and facilitates oncogenic activities	[68]
Inducing proliferative signaling and enhancing exosome biogenesis via increasing neutral sphingomyelinase 2 activity	Enhancement	HBV	HBx protein and mRNA encapsulated within exosomes	Altered exosomal cargo (quantitatively and qualitatively) and promote HBV-associated liver diseases	[68]
Induce mRNA expression of the NKG2D ligand in macrophages	Inhibition	HBV	Exosomes generated from HBV infected cells and which contain viral RNA	NK cell activation, confirmed by CD69 upregulation, and induction of IFN-γ production promoting innate immunity and lymphocyte activation to defend the host from infections	[69,70]
Infection with HBV	Enhancement	HBV	Exosomes generated from HBV infected cells	An increase in immunosuppressive miRNAs: miR-21 and miR-29a, within CD81+ exosomes, transferred from hepatocytes to macrophages	[69]
Downregulation of IL-12p35 and IL-12p40	Enhancement	HBV	Exosomes generated from HBV infected cells and containing immunosuppressive miRNAs: miR-21 and miR-29a	Potential inhibition of NK cell activity and facilitation of viral evasion of the host immune response	[69]
HBV modulation of exosome-bound proteins, including the increase of 5 proteasome subunit proteins: PSMD1, PSMD7, PSMD14, PSMC1, and PSMC2, enhancing proteolytic activity	Enhancement	HBV	35 exosome-bound proteins quantitatively altered as a result of HBV infection in HBV-infected HepAD38 hepatoblastoma cell line-derived exosomes	Significant reducing monocyte IL-6 production and modulation of proinflammatory molecules	[71]
Uptake of these HBV-laden exosomes by cells	Enhancement	HBV	HBV-laden exosomes	Impairment of NK cell production of IFN-γ, NK cell survival and proliferation, cytolytic activity, and NK cell responsiveness to stimulation from poly (I:C)	[72]
Antiviral activity has been observed to be transferred from liver nonparenchymal cells (LNPCs) to hepatocytes via exosomes	Inhibition	HBV	LNPC-derived exosomes	IFN-α induced HBV antiviral activity	[73]
**Flaviviridae**					
Viral packaging within vesicles.	Enhancement	HCV	Exosome-bound viral particles	Increased viral spread. Activate immune cells and establish infection	[74,75]
Transportation of viral regulatory elements: Human Ago2 and miR-122	Enhancement	HCV	Exosome-bound Ago2 and miR-122	Increased viral spread	[74,76]
Infected-tick cell-derived EVs mediate transmission of viral RNA and NS1 protein	Enhancement	LGTV	Exosome-bound viral RNA and NS1	Increased transmission from arthropod vectors to humans. Disseminate virus within host neuronal cells.	[31,77]
Transfer of antiviral properties from EVs carrying C19MC miRNAs	Inhibition	ZIKV	EV-bound C19MC miRNAs	Increased autophagy and viral resistance. Decreased ZIKV viral replication.	[83,84]
Downregulation of miR-21 after exposure to EVs	Inhibition	ZIKV	Infected HPT cell-derived EVs	Decreased TLR7-mediated neurotoxicity	[87,88]
Exposure of placental cells to EVs	Enhancement	ZIKV	Macrophage-derived exosomes	Induction of placental proinflammatory cytokine production.	[91]
Stimulation of human macrophage IL-1β secretion	Enhancement	ZIKV	ZIKV NS5-mediated activation of NLRP3	Activation of host inflammatory response and macrophage recruitment promotes inflammation	[92]
EVs transmitted across neurons	Enhancement	ZIKV	EV-bound ZIKV-RNA and E-protein	Increased ZIKV transmission across neurons	[93]
Modulation of SMPD3 activity as a result of ZIKV cortical neuron infection	Enhancement	ZIKV	EV-bound SMPD3	Increased EV biogenesis, viral burden, and viral transmission	[93]
**Retrovirdae**					
Release of HIV-1 infected cell-derived EVs	Enhancement	HIV	gp120 laden HIV-1 envelope (Env) protein	Increased HIV-1 infectivity in lymphoid tissues	[119,120,121,122]
Increased EV-mediated Nef egress	Enhancement	HIV	EV-bound Nef protein	Increased EV secretion, presence of MVBs within cells, decay of CD4+ T-cell populations	[123,124,125,126]
Transport of Nef via exosomes to target cells	Enhancement	HIV	EV-bound Nef protein	Promote decay of CD4+ T-cell populations, promote CD8+ T-cell activity, CXCR4-mediated apoptosis, and ADAM17 activation increasing CD4+ T-cell permissiveness to HIV-1	[127,128,129,130]
Differential miRNA content relative to uninfected cells	Enhancement	HIV	HIV-1 co-evolution with the host	Facilitating suppression of host RNA interference (RNAi)	[131,137]
Release of HIV-1 infected plasma and macrophage derived EVs	Enhancement	HIV	HIV-1-derived miRNAs, vmiR88 and vmiR99	Promoting macrophage release of TNF-α, thus supporting chronic immune activation	[138]
Modulation of exosomal and cellular miRNA profiles	Enhancement	HIV	EV-bound Nef protein	Modulation of HIV-1 pathogenesis and viral replication	[139]
Reduced ZO-1 TJ protein expression in HBMECs and increasing TLR-induced chemokines and cytokines in microglia	Enhancement	HIV	Microglia-derived EV-bound Nef	Disruption of BBB permeability and integrity	[141]
**Coronaviridae**					
Uptake of these SARS-CoV-2 exosomes by human induced pluripotent stem cell-derived cardiomyocytes (hiPSC-CMs)	Enhancement	SARS-CoV-2	SARS-CoV-2 infected cell-derived exosomes	Upregulation of genes associated with inflammation in hiPSC-CM	[133]
Delivery of viral RNA packaged within exosomes	Enhancement	SARS-CoV-2	SARS-CoV-2 infected cell-derived exosomes	Indirect infection of target cardiomyocytes, exacerbating pathology, and altering the inflammatory state	[134]
Incorporation of spike S protein into exosomes and priming with the S-protein exosome vaccine with subsequent boosting via addition of adenoviral vector vaccine	Inhibition	SARS-CoV-2	Vaccine: Exosomes incorporated with spike S proteins	Generation of neutralizing antibodies titers exceeding those of a SARS-convalescent patient serum	[136,142]
**Pathogenesis**	**Component**	**Outcome**	**Component**	**Outcome**	**Reference**
**Retrovirdae**					
CD8+ T-cell derived exosome transport	Inhibition	HIV	Membrane bound anti-HIV protein moiety	Decreased HIV-1 replication	[101]
Transport of antiviral factors at both the protein and mRNA level	Inhibition	HIV	TLR3-activated HBMEC-derived exosomes-bound antiviral factors	Block HIV-1 infection to the CNS. Transferring anti-HIV protection to macrophages	[102]
Release of TLR3-activated IEC-derived exosomes containing anti-HIV-1 factors	Inhibition	HIV	HIV-restriction miRNAs (miRNA-20 and miRNA125b), and IFN-stimulated genes (ISGs: ISG15, OAS-1, and Viperin)	Increased Anti-HIV GI innate immunity	[103]
Blocking viral reverse transcription	Inhibition	HIV	Vaginal fluid-derived EVs	Post-entry block of HIV-1 replication	[105]
Deleterious effects upon HIV-1 reverse transcriptase activity	Inhibition	HIV	Non-infected semen-derived EVs	Post-entry block of HIV-1 replication	[105,106]
Viral packaging within vesicles.	Enhancement	HIV	Semen-derived EV-bound functional viral mRNA	Increased viral spread	[105]
Binding to DC-SIGN receptor, competing with HIV-1	Inhibition	HIV	Uninfected donor breast milk-derived EVs	Decreased HIV-1 infection of DC and viral transfer to CD4+ T-lymphocytes	[107]
Transport of PBMC-derived EVs to neighboring cells deficient in CCR5	Enhancement	HIV	PBMCs-derived EVs containing CCR5	Enhanced cellular susceptibility to HIV-1	[109]
Delivering the HIV-1 co-receptor to nearby tissues lacking CXCR4 expression	Enhancement	HIV	Megakaryocyte-derived EVs containing CXCR4	Facilitates viral spread	[110,111]
Transport of EVs to endothelial cells and PBMCs deficient in CCR5	Enhancement	HIV	PBMC-derived EV-encapsulated CCR5 chemokine receptors	Enhancing HIV-1 infection	[109]
Transport of EVs to cells deficient in CXCR4	Enhancement	HIV	Megakaryocyte and platelet-derived EV-encapsulated CXXR4 receptors	Enhancing HIV-1 infection	[110,111]
Binding of EV-bound TIM-4 to HIV-1 PS surface-bound moieties	Enhancement	HIV	EV-bound TIM4 receptor	Increased exosome-mediated trafficking of HIV-1 to human immune cells	[112,113]
HIV-1 Entrapping itself with exosome aggregates via exploitation of exosomal surface properties	Enhancement	HIV	Exosomal surface properties	Host-immune system evasion via camouflage. Increased viral spread	[116]
Exposure of EVs to macrophages yield a significant rise in proinflammatory cytokines, TNF-β, and IL-6	Enhancement	HIV	HIV-1 infected primary cell-derived EV-bound TAR	Enhance undifferentiated naïve cell susceptibility to HIV-1 infection	[117]
DC-CD44 receptor binding of apoptotic microvesicles	Enhancement	HIV	Apoptotic body-bound DC-CD44 receptor	Decreased DC-dependent cytokine production and inhibition of DC-mediated T/NK-cell priming	[3]
